# Common biomarkers of idiopathic pulmonary fibrosis and systemic sclerosis based on WGCNA and machine learning

**DOI:** 10.1038/s41598-024-84820-3

**Published:** 2025-01-03

**Authors:** Ning Shan, Yu Shang, Yaowu He, Zhe Wen, Shangwei Ning, Hong Chen

**Affiliations:** 1 Harbin Medical University, Harbin, Heilongjiang Province, China; 2 Department of Pulmonary and Critical Care Medicine, The Second Affiliated Hospital of Harbin Medical University, Harbin, Heilongjiang Province, China; 3 The Second Hospital of Heilongjiang Province, Harbin, Heilongjiang Province, China; 4 College of Bioinformatics Science and Technology, Harbin Medical University, Harbin, Heilongjiang Province, China

**Keywords:** Idiopathic pulmonary fibrosis, Systemic sclerosis, WGCNA, GSEA, scRNA sequencing analysis, CCL2, Data mining, Data processing, Machine learning, Chemokines

## Abstract

Interstitial lung disease (ILD) is known to be a major complication of systemic sclerosis (SSc) and a leading cause of death in SSc patients. As the most common type of ILD, the pathogenesis of idiopathic pulmonary fibrosis (IPF) has not been fully elucidated. In this study, weighted correlation network analysis (WGCNA), protein‒protein interaction, Kaplan–Meier curve, univariate Cox analysis and machine learning methods were used on datasets from the Gene Expression Omnibus database. CCL2 was identified as a common characteristic gene of IPF and SSc. The genes associated with CCL2 expression in both diseases were enriched mainly in chemokine-related pathways and lipid metabolism-related pathways according to Gene Set Enrichment Analysis. Single-cell RNA sequencing (sc-RNAseq) revealed a significant difference in CCL2 expression in alveolar epithelial type 1/2 cells, mast cells, ciliated cells, club cells, fibroblasts, M1/M2 macrophages, monocytes and plasma cells between IPF patients and healthy donors. Statistical analyses revealed that CCL2 was negatively correlated with lung function in IPF patients and decreased after mycophenolate mofetil (MMF) treatment in SSc patients. Finally, we identified CCL2 as a common biomarker from IPF and SSc, revealing the common mechanism of these two diseases and providing clues for the study of the treatment and mechanism of these two diseases.

## Introduction

IPF is defined as chronic, progressive ILD that primarily occurs in older people; the median survival time after diagnosis is approximately 2–3 years, and its pathogenesis is still unclear^1^. The BRIC countries (Brazil, Russia, India, and China) may include 1 million cases of IPF^2^. For IPF management, pirfenidone and the triple tyrosine kinase [vascular endothelial growth factor (VEGF), fibroblast growth factor (FGF) and platelet-derived growth factor (PDGF)] inhibitor nintedanib are recommended to delay the progression of fibrosis, which cannot be reversed^3^. Moreover, lung transplantation is considered the final method for patients with moderate to severe disease^4^.

Current studies suggest that the pathogenesis of IPF may involve transforming growth factor-β (TGF-β), which is increased in patients with pulmonary fibrosis^5,6^. VEGF can improve pulmonary hypertension (PH), but simultaneously aggravates pulmonary fibrosis, which may play opposite roles in different lung compartments^7^.

SSc, also known as scleroderma, is a type of autoimmune connective-tissue disease (CTD), with the clinical presentation of hard, thickened areas of skin, Raynaud’s phenomenon, and gastroesophageal reflux, and has the highest mortality rates of all rheumatic diseases^8,9^. The pathogenesis of SSc varies, is complex, has not been fully elucidated, and may be related to genetics, the environment, disorders of the immune response and vascular lesions^10^. The prevalence in Europe ranges from 7.2 to 33.9 per 100,000 individuals^11,12^. Patients with SSc are often difficult to diagnose early in the course of the disease, delaying their appropriate treatment and care^13^.

With respect to pathogenesis, some studies have proposed that the expression of TGF-β, TGF-βR1 and TGF-βR2 is increased in the skin cells of SSc patients, indicating that TGF-β may play a role in SSc skin lesions^14^. The level of VEGF in the plasma of SSc patients increases with increasing clinical stage. It is believed to be involved in the disease progression of SSc and in the remodelling of the skin microvasculature by hypoxia-induced endothelial‒mesenchymal transition (EndoMT)^15,16^. For the management of SSc patients, methotrexate and MMF are recommended for skin-associated complications^17,18^.

The pathogenesis and metabolic pathways of IPF and SSc are related. CCL2, a chemokine, has been shown to be involved in the pathogenesis of IPF by being regulated by the FOXF1/R-Ras signalling, NFATc3 and in the pathogenesis of SSc by being a downstream signalling molecule with low NCF1 activity^19–21^. The pathogenesis of these two diseases has not been fully elucidated, and existing treatments are not curable, placing a large burden on patients and health care systems. Therefore, the need to study the common markers and molecular pathways of IPF and SSc is urgent. In this study, WGCNA and machine learning were used to select markers related to the disease status of these 2 diseases and the prognosis of IPF, and the markers were then analysed via multiple methods, providing a possible direction for the pathogenesis and treatment of IPF and SSc.

## Results

### Removal of batch effects and PCA

**Fig. 1:**
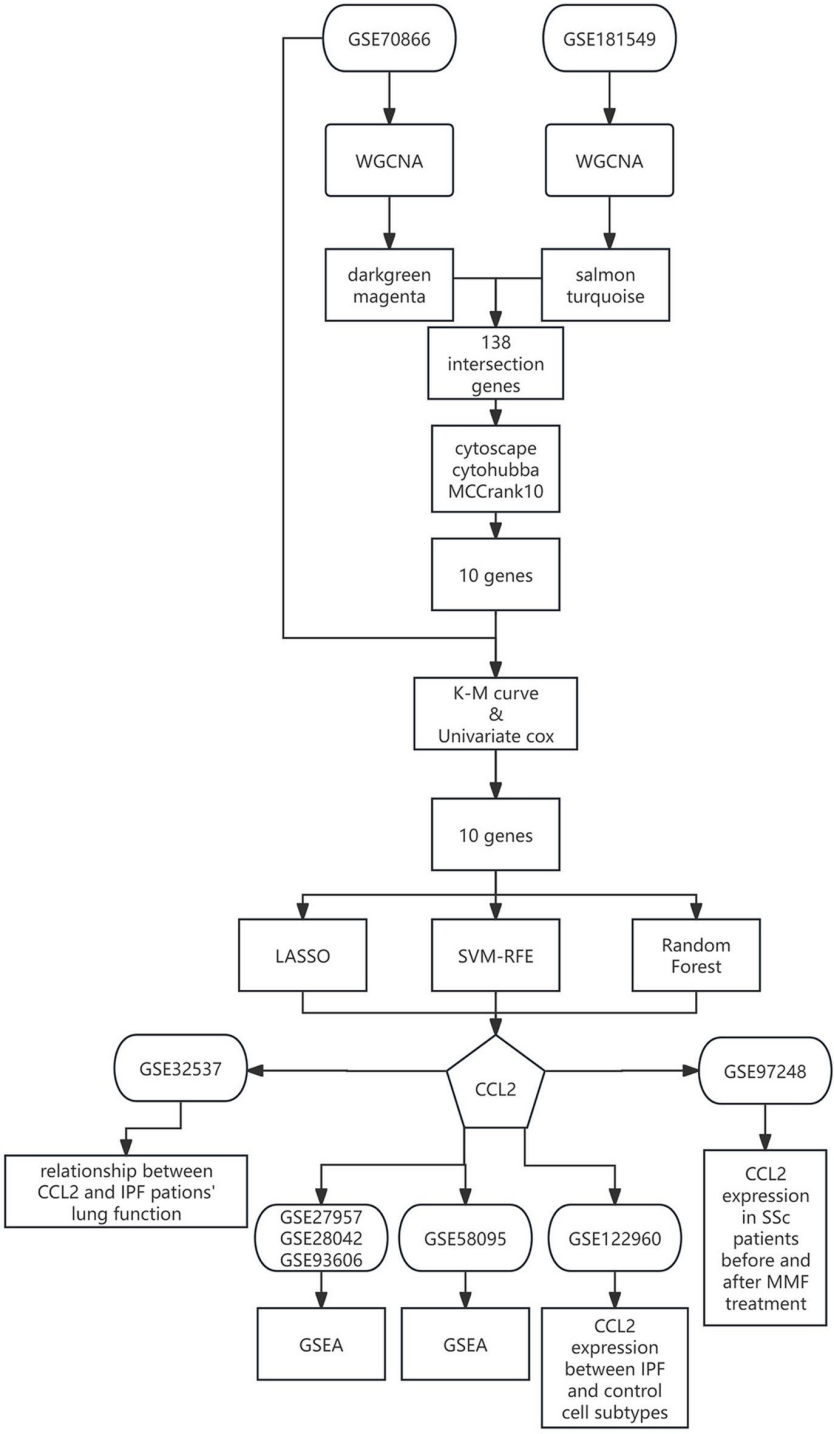
Flow chart of the study.

**Fig. 2:**
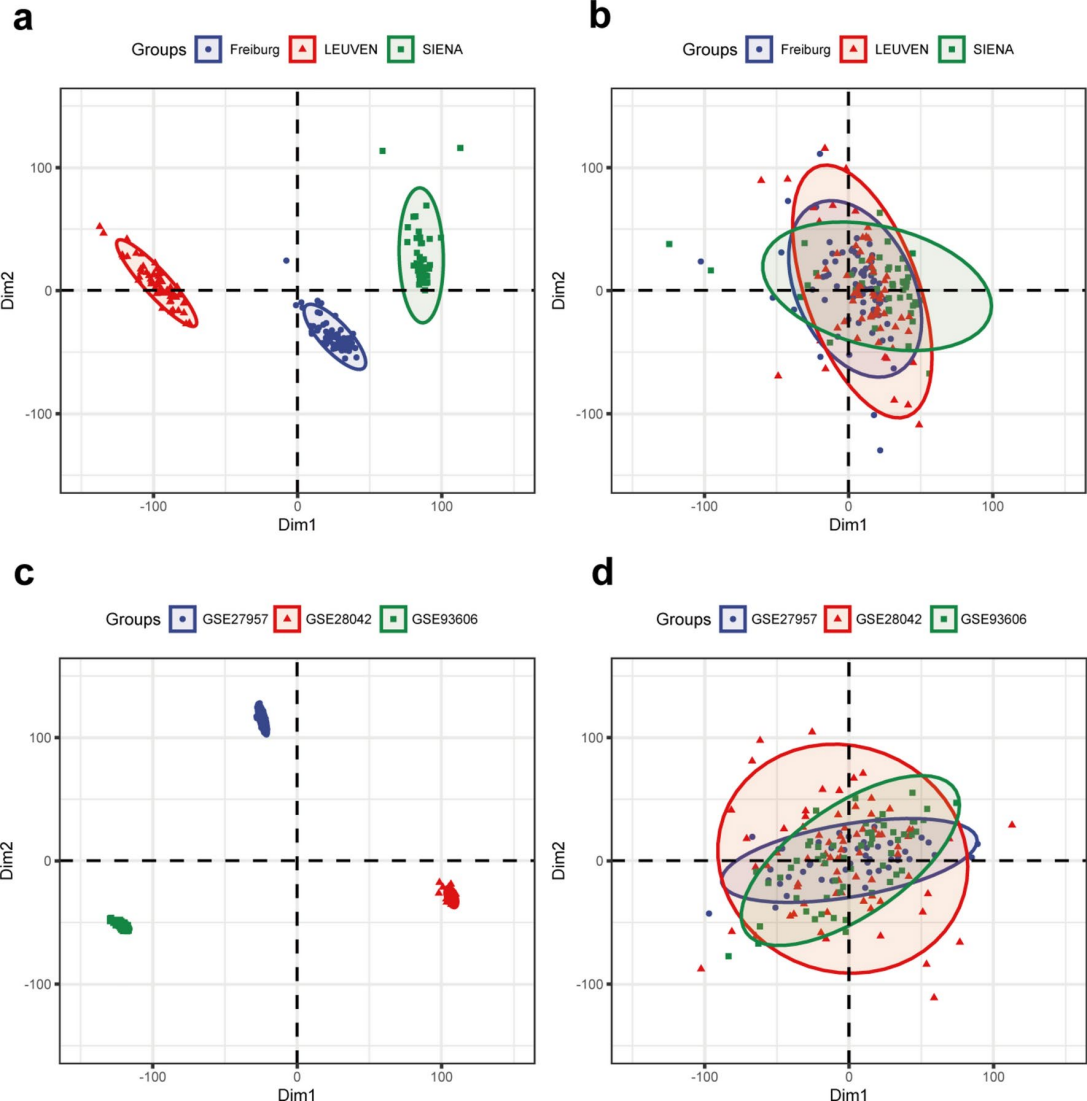
Principal Component Analysis (PCA) of IPF datasets before and after batch correction and normalization. (**a**,**b**) PCA of 3 cohorts in GSE70866 before and after batch correction and normalization. (**c**,**d**) PCA of baseline IPF samples in GSE27957, GSE28042 and GSE93606 before and after batch correction and normalization.

The research flowchart of this research was shown in Fig. 1. Batch effects were eliminated between the 3 cohorts in GSE70866 (Fig. 2a,b) and the baseline IPF samples in GSE27957, GSE28042, and GSE93606 (Fig. 2c,d).

### WGCNA, PPI, survival analysis and machine learning were used to identify CCL2 as a common characteristic gene for IPF and SSc diagnosis and the prognosis of IPF

**Fig. 3:**
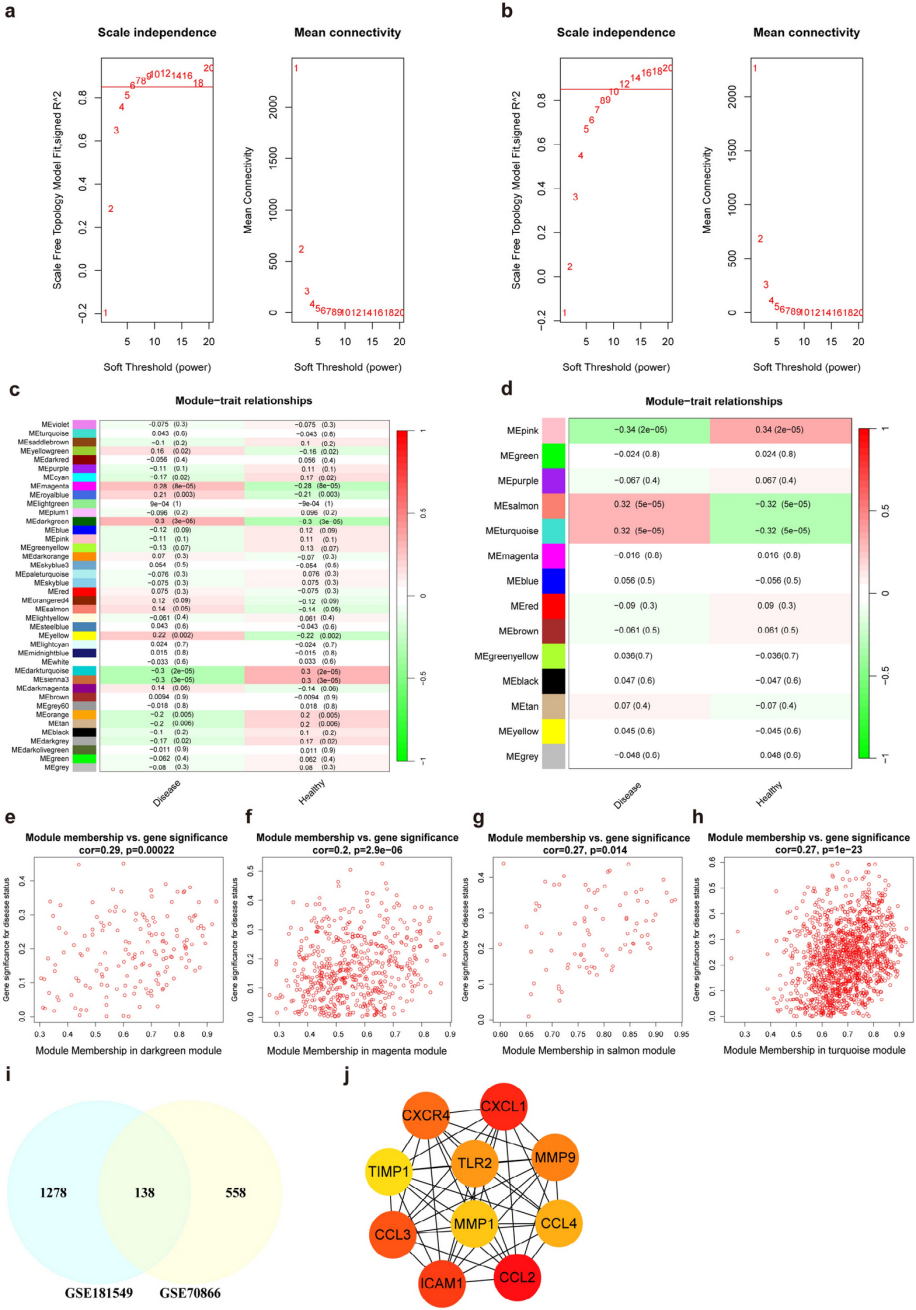
Weighted gene co-expression network analysis for identification and analysis of characteristic genes common to the IPF and SSc datasets. (**a**,**b**) The left panel shows the scale-free fitting exponent analysis with multiple soft threshold powers (β), and the right panel shows the average connectivity analysis with multiple soft threshold powers. Figure a is for the IPF dataset, and figure b is for the SSc dataset. (**c**,**d**) Heatmap showing the relationships between module eigengenes and IPF or SSc status. The correlation (left) and p-value (right) of the module feature genes with disease status are shown. Figure c shows the IPF dataset, and figure d shows the SSc dataset. (**e**–**h**) The correlation plot between the module membership and the gene significance of genes in each module. (**e**,**f**) correspond to the darkgreen and magenta modules in the IPF dataset, respectively, (**g**,**h**) correspond to the salmon and turquoise modules in the SSc dataset, respectively. (**i**) The intersection of genes in key modules of two diseases was obtained via a Venn diagram. (**j**) Based on the MCC method of the Cytoscape plug-in CytoHubba, PPI network analysis was performed for the top 10 genes among the 138 intersecting genes.

As shown in Fig. 3a–h, we performed WGCNA for GSE70866 and GSE181549 to identify the module most strongly correlated with IPF and SSc. Figure 3a,c,e,f are for GSE70866, Fig. 3b,d,g,h. are for GSE181549.

For GSE70866, β = 6 (scale-free R^2^ = 0.85) was chosen as the “soft” threshold (Fig. 3a). Then, according to Fig. 3c, we chose darkgreen module and magenta module as the modules that were most strongly correlated with the IPF. In addition, we found that there was a strong association between module membership and gene significance in these 2 modules (Fig. 3e,f).

For GSE181549, we chose β = 12 (scale-free R^2^ = 0.85) as the “soft” threshold (Fig. 3b). On the basis of Fig. 3d, the salmon module and turquoise module were chosen as the modules that were most strongly correlated with SSc. Moreover, a strong association was found between module membership and gene significance in these 2 modules (Fig. 3g,h).

The intersection of the above two pairs of most correlated modules contained 138 genes (Fig. 3i). To determine the relationship between potential pathogenic genes common to IPF and SSc, the 138 intersection genes were input into the STRING database (https://www.string-db.org/) with a medium confidence score of > 0.4. These common potential pathogenic genes were visualized via Cytoscape software, and the top rank 10 genes were identified via maximum clique centrality (MCC) methods (Fig. 3j).

**Fig. 4:**
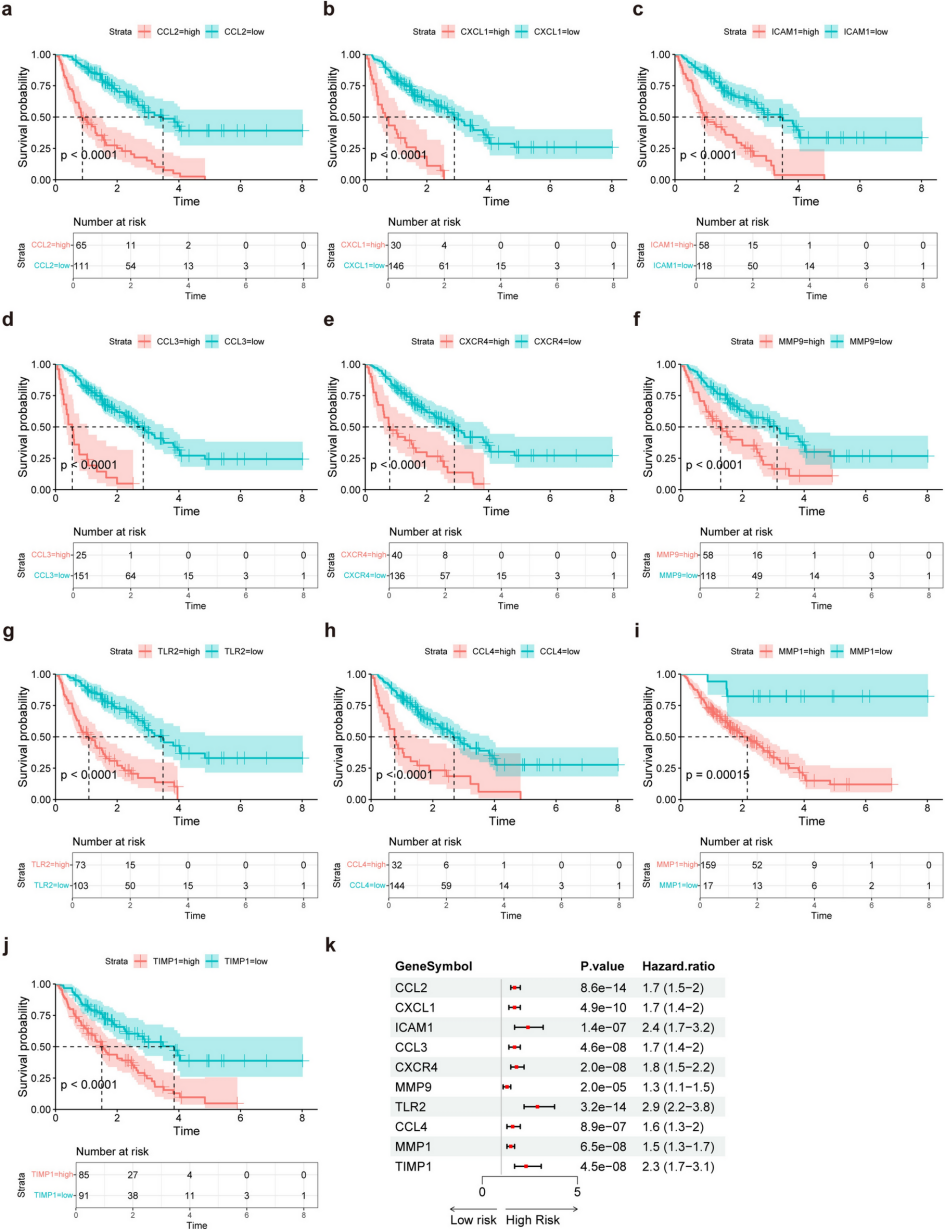
Survival analysis of the influence of 10 intersecting genes on the prognosis of IPF. (**a**–**j**) K–M curves of the influence of 10 intersecting genes on the prognosis of IPF, a to j represent CCL2, CXCL1, ICAM1, CCL3, CXCR4, MMP9, TLR2, CCL4, MMP1 and TIMP1, respectively. (**k**) Forest plot of univariate Cox analysis of the effects of 10 intersecting genes on IPF prognosis.

K–M curves of these 10 genes were plotted on the basis of the prognostic information of the IPF samples from GSE70866, which revealed that all 10 genes were risk factors for the prognosis of IPF (Fig. 4a–j). Univariate Cox regression revealed that the hazard ratios (HRs) of these 10 genes were all greater than 1, indicating that these 10 genes are risk factors for the prognosis of IPF (Fig. 4k).

**Fig. 5:**
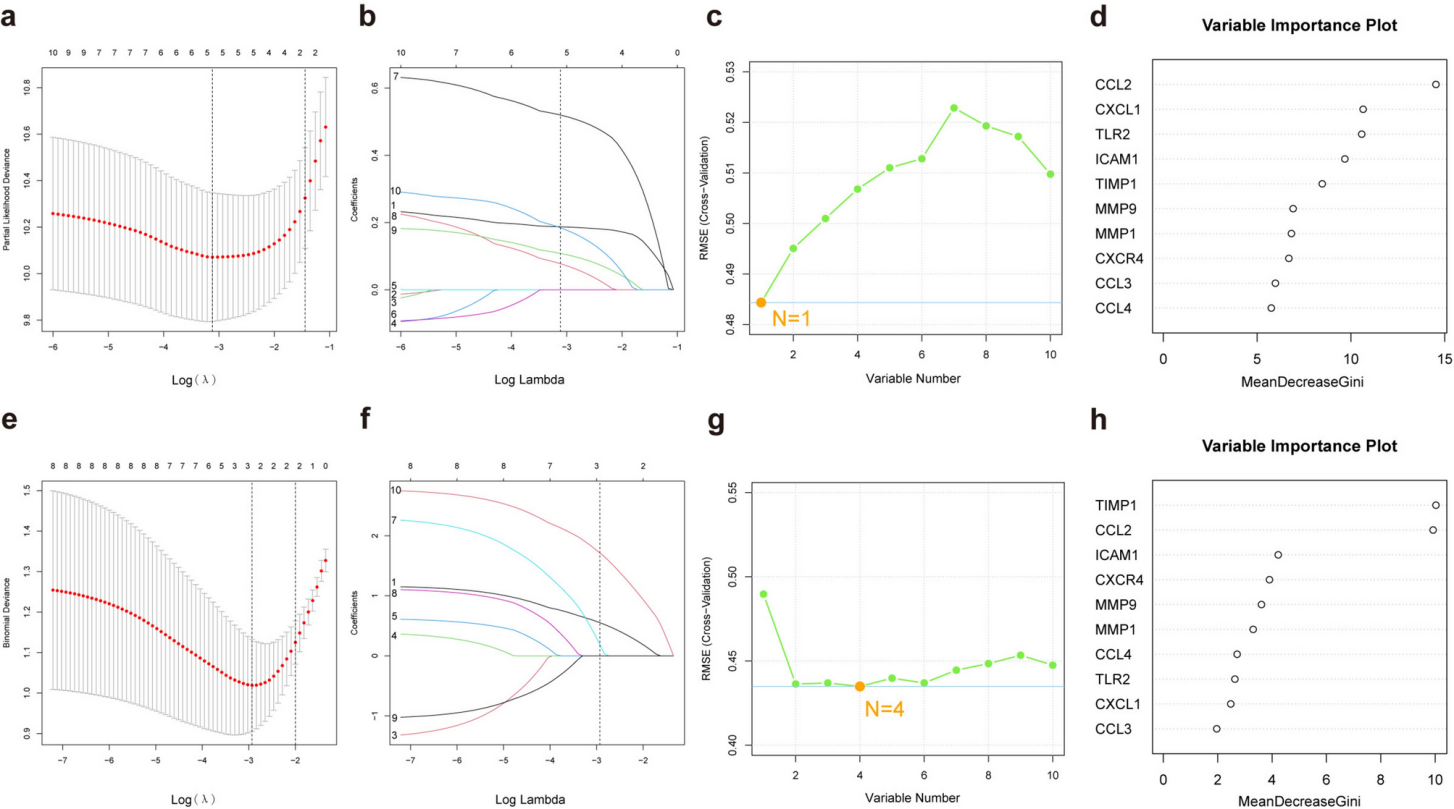
Machine learning was used to screen characteristic genes for IPF prognosis and SSc diagnosis. (**a**,**b**) Biomarker screening in LASSO-cox models. The number of genes corresponding to the lowest point of the curve (n = 5) was most suitable for assessing the prognosis of IPF. (**c**,**g**) Screening for biomarkers via the SVM-RFE algorithm. (**d**,**h**): The random forest method was used to screen biomarkers, sequencing the importance of genes according to MeanDecreaseGini values. (**e**,**f**) Biomarker screening in LASSO models. The number of genes corresponding to the lowest point of the curve (n = 3) was most suitable for assessing the diagnosis of SSc.

Several machine learning algorithms were then applied to both the merged dataset of baseline IPF samples in GSE70866 and the SSc samples in GSE58095. As shown in Fig. 5a,b, Least Absolute Shrinkage and Selection Operator (LASSO)-cox algorithm identified 5 potential biomarkers, and as illustrated in Fig. 5c, Support Vector Machines-Recursive Feature Elimination (SVM-RFE) algorithm revealed that a model involving 1 gene achieved the lowest Root Mean Square Error (RMSE). Moreover, 3 genes were identified via the random forest algorithm (Fig. 5d). By intersecting the results of these 3 algorithms, we defined CCL2 as the common characteristic gene in IPF prognosis. As shown in Fig. 5e,f, the LASSO algorithm defines 3 biomarkers associated with SSc diagnosis. Figure 5g shows that SVM-RFE indicates that the 4-gene model has the lowest RMSE. In addition, the random forest results revealed that 2 biomarkers had higher Mean Decrease Gini values (Fig. 5h). From the intersection of the results of these 3 machine learning algorithms, we selected TIMP1 and CCL2 as biomarkers for SSc diagnosis. In summary, CCL2 was selected as the biomarker most strongly associated with IPF prognosis and the diagnosis of both IPF and SSc.

### GSEA enrichment analysis

**Fig. 6:**
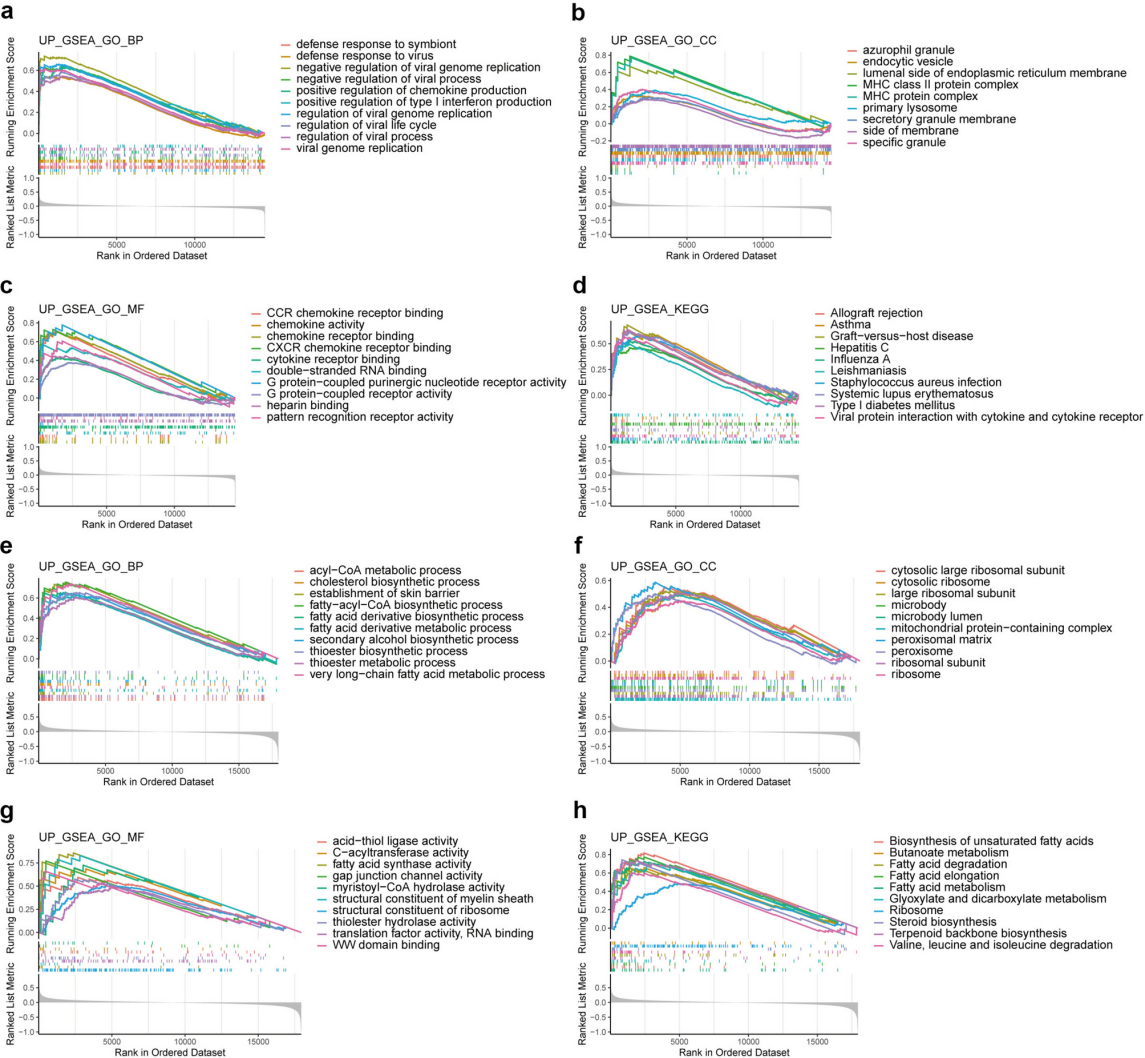
GSEA based on the CCL2 expression level. (**a**–**d**) are for the IPF dataset, (**e**–**h**) are for the SSc dataset. (**a**,**e**) GO: BP analysis via GSEA. (**b**,**f**) GO: CC analysis via GSEA. (**c**,**g**) GO: MF analysis via GSEA. (**d**,**h**) KEGG analysis via GSEA.

The genes up-regulated with IPF patients’ CCL2 expression value was mainly enriched in immune related components, especially the chemokine family (Fig. 6a,c), such as positive regulation of chemokine production, CCR chemokine receptor binding, chemokine activity, chemokine receptor binding, CXCR chemokine receptor binding and cytokine receptor binding. Other components and pathways that are positively associated with CCL2 granule in IPF patients include azurophil granule and several immune-related pathways, such as allograft rejection, asthma, graft-versus-host disease and viral protein interaction with cytokine and cytokine receptor (Fig. 6b,d).

In the SSc dataset, the gene up-regulated with CCL2 expression value was enriched mainly in lipid metabolism, such as acyl − CoA metabolic process, cholesterol biosynthetic process, establishment of skin barrier, fatty − acyl − CoA biosynthetic process, fatty acid derivative biosynthetic process, fatty acid derivative metabolic process, mitochondrial protein-containing complex, fatty acid synthase activity, biosynthesis of unsaturated fatty acids, butanoate metabolism, fatty acid degradation, fatty acid elongation and fatty acid metabolism (Fig. 6e–h). We found that these genes are involved mainly in fatty acid metabolism.

### Common characteristic gene’s expression in single cells

**Fig. 7:**
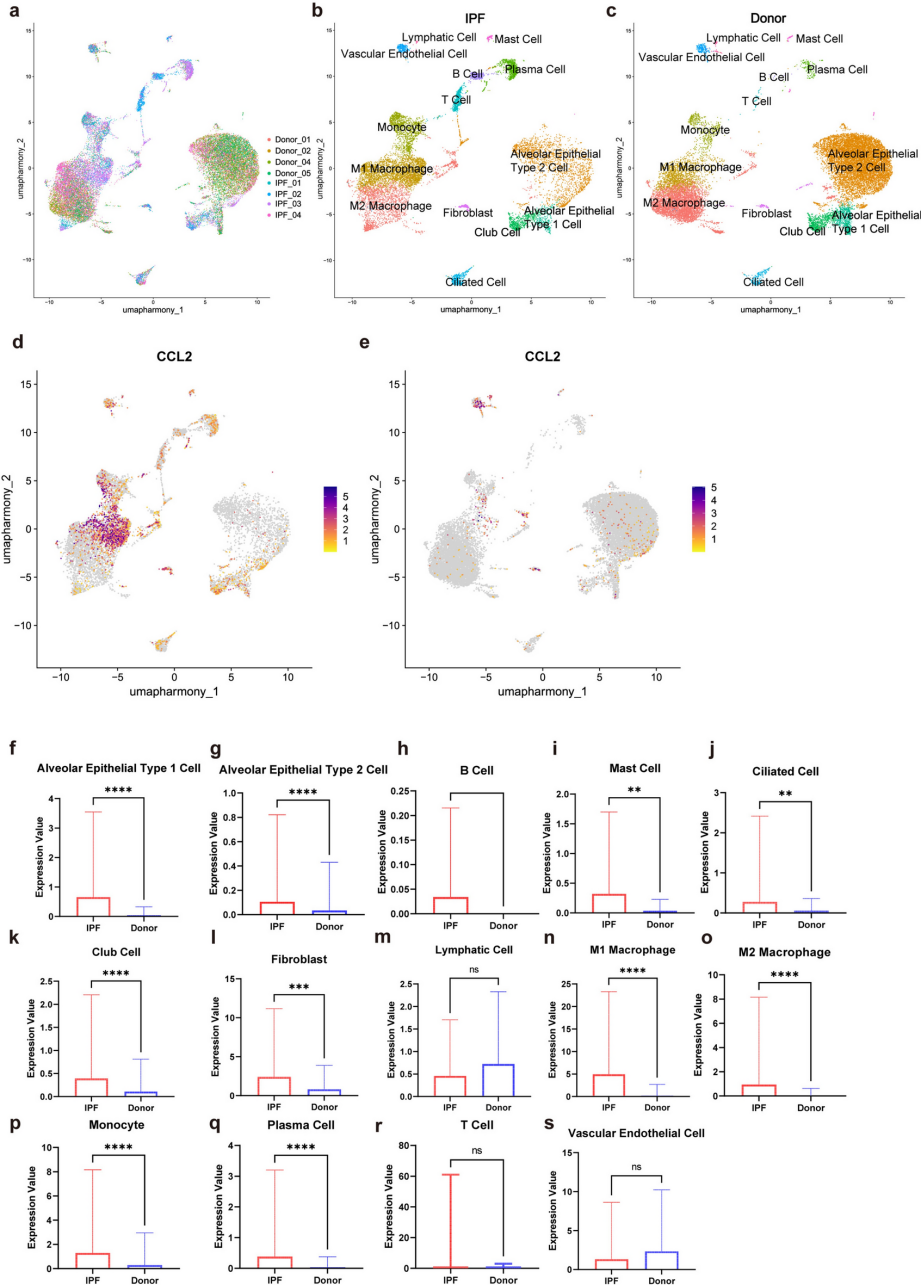
The expression profile of common characteristic genes in IPF single cells and the difference in CCL2 gene expression between IPF patients and healthy controls. (**a**) UMAP of 8 samples after removal of the batch effect. (**b**) Cellular subtypes of IPF lungs. (**c**) Cellular subtypes of healthy donor lungs. (**d**) CCL2 gene expression in IPF lungs. (**e**) CCL2 gene expression in healthy donor lungs. (**f**–**s**) Histogram of the mean expression value of CCL2 in each cell subtype of IPF and healthy control samples, with the standard deviation (SD). *p < 0.05; **p < 0.01; ***p < 0.001; ****p < 0.0001; ns not significant.

4 IPF samples and 4 healthy samples were chosen for single-cell analysis (Fig. 7a). All the cells in these 8 samples’ UMAP were annotated into 14 categories: Alveolar Epithelial Type 1 Cell [number of cells in IPF group (n1) = 250, number of cells in Healthy Donor group (n2) = 553], Alveolar Epithelial Type 2 Cell (n1 = 2168,n2 = 10,089), B Cell (n1 = 412,n2 = 11), Mast Cell (n1 = 137,n2 = 81), Ciliated Cell (n1 = 397, n2 = 209), Club Cell (n1 = 596,n2 = 226), Fibroblast (n1 = 137,n2 = 135), Lymphatic Cell (n1 = 35,n2 = 86), M1 Macrophage (n1 = 3183,n2 = 1645), M2 Macrophage (n1 = 2212,n2 = 5230), Monocyte (n1 = 1424,n2 = 537), Plasma Cell (n1 = 886,n2 = 146), T Cell (n1 = 611,n2 = 35), Vascular Endothelial Cell (n1 = 245,n2 = 257) (Fig. 7b,c). The expression values of the common characteristic gene CCL2 in IPF and healthy samples were compared (Fig. 7d,e), and Mann–Whitney U test was conducted based on the fact that the expression data of all cell types did not follow the normal distribution. Then we found significant differences in CCL2 expression in Alveolar Epithelial Type 1 Cell (*p* < 0.0001) , Alveolar Epithelial Type 2 Cell (*p* < 0.0001), Mast Cell (*p* = 0.0088), Ciliated Cell (*p* = 0.0023), Club Cell (*p* < 0.0001), Fibroblast (*p* = 0.0001), M1 Macrophage (*p* < 0.0001), M2 Macrophage (*p* < 0.0001), Monocyte (*p* < 0.0001) and Plasma Cell (*p* < 0.0001), and the tests were all two-tailed (Fig. 7f–s). Because the n2 value of B cells was too small (n2 = 11) and CCL2 expression was 0 in the healthy donor group, the Mann–Whitney U test did not yield a p value and was not included in subsequent analyses.

### CCL2 expression was negatively correlated with lung function in patients with IPF and decreased after MMF treatment in diffuse cutaneous systemic sclerosis (dcSSc) patients

**Fig. 8:**
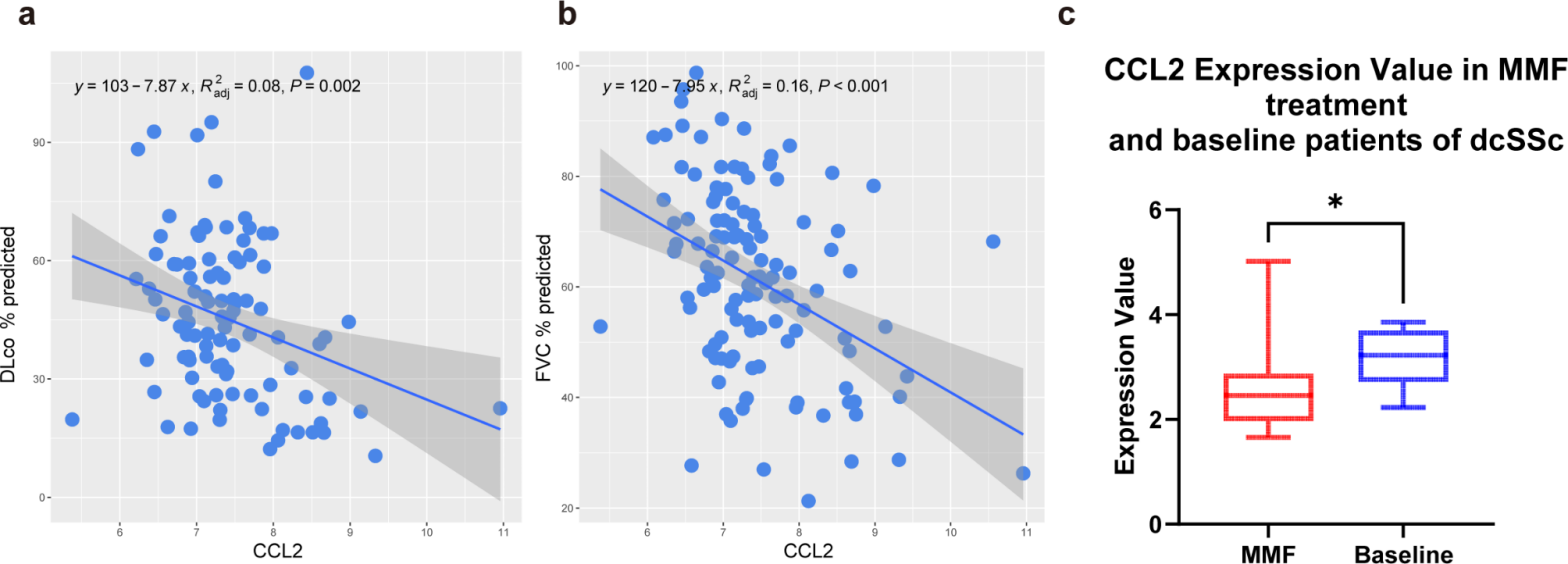
Scatterplot of the relationship between lung CCL2 expression and lung function in patients with IPF. (**a**) Relationship between CCL2 expression and DLco % predicted in lung tissue. (**b**) Relationship between CCL2 expression and FVC % predicted in lung tissue. (**c**) Box plot of CCL2 expression in the skin tissue of SSc patients before and after treatment with MMF. *p < 0.05.

The relationship between IPF patients’ CCL2 expression and their lung function [diffusion capacity of carbon monoxide in the lung (DLco) % predicted and forced vital capacity (FVC) % predicted)] according to GSE32537 is shown in Fig. 8a,b. With increasing CCL2 expression value, DLco % predicted and FVC % predicted decreased in IPF patients. A Wilcoxon matched-pairs signed rank test was performed on the dcSSc samples before and after the use of MMF in GSE97248 (n = 18 in each group). After MMF treatment for 3 months, CCL2 expression was significantly lower than before (*p* = 0.0133) (Fig. 8c).

## Discussion

As a systematic biological method, WGCNA can reveal the correlation patterns of gene expression between different samples and identify gene co-expression modules related to phenotypes. Compared with differentially expressed genes, WGCNA not only studies the relationships between genes and phenotypes but also focuses on the interactions between genes, providing an important method for screening disease-related genes. In our study, the WGCNA algorithm was used to identify the co-expressed modules in IPF and SSc, and then, in order to identify potential closely interacting protein-coding genes in the intersection, thereby screening genes that play more important roles in IPF and SSc, PPI was constructed on the basis of the 138 intersection genes. To more visually examine and describe the relationship between the incidence of endpoint events (deaths) and the corresponding gene expression levels in IPF patients, we performed survival analyses, including K–M curves and univariate Cox analysis. After survival analysis and machine learning for the 10 genes in the PPI network, the CCL2 gene was identified as an important biomarker that plays a key role in the pathogenesis of IPF and SSc.

CCL2, also known as monocyte chemoattractant protein-1 (MCP-1), was the first CC chemokine to be discovered and studied; it preferentially binds to its receptor, CCR2, a G-protein-coupled 7 transmembrane receptor^22,23^. The TGF-β pathway plays an important role in almost all types of fibrosis and involves multiple signalling cascades^24^. A previous study revealed that TGF-β1 induces fibroblasts to produce CCL2, which can then induce further fibrotic responses in these cells, our result in Fig. 7l also shows that compared with healthy control, CCL2 expression level is higher in the fibroblast of IPF patients, indicating that the TGF-β pathway might be activated in IPF patients^25^. The study also revealed that CCL2 is downstream of TGF-β1-induced gene expression, which indicates that CCL2 is a member of the TGF-β pathway^25^. Paôline reported that in SSc patients, CCL2 can be produced at high levels by DC-SIGN- positive alternatively activated macrophages, a type of macrophage related to the degree of skin fibrosis in SSc patients, indicating that CCL2 is involved in the vicious cycle of skin fibrosis in SSc patients^26^. Another study revealed that the serum level of CCL2 in SSc patients is significantly greater than that in controls and is positively correlated with the modified Rodnan skin score (mRSS), confirming the role of CCL2 in extracellular matrix (ECM) deposition^27^.

Karman showed that in the myeloid-enriched IPF subset, the monocyte‒macrophage chemoattractant axis, which potentially includes the CCL2‒CCR2 axis, was highly activated, and this axis might play a role in recruiting inflammatory macrophages to this subset^28^. Our GSEA results revealed that the genes upregulated in IPF patients with high CCL2 expression were enriched in monocyte chemotaxis, positive regulation of chemokine production, CCR chemokine receptor binding, chemokine activity and chemokine receptor binding. We speculate that in patients with IPF, the chemokine system is widely activated and might be a target for the treatment of IPF.

Chemokines were initially found to be mediators of the targeted migration of immune cells to sites of inflammation and injury, including 4 major subfamilies, CC chemokines, CXC chemokines, CX3C chemokines and XC chemokines, and each of these subfamilies has its own receptor: CCRs, CXCRs, CX3CRs and XCRs^29^. Our survival study revealed several chemokines and chemokine receptors, including CCL2, CCL3, CCL4, CXCL1 and CXCR4. All of them are risk factors for the prognosis of IPF. Figure 3j also revealed a strong interaction between these chemokines and chemokine receptors, suggesting that chemokines play a pivotal role in IPF pathogenesis and might be predictors of the prognosis of IPF, especially CCL2. Figure 8a,b also revealed that the CCL2 expression level in IPF patients is negatively correlated with their lung function (DLco % predicted and FVC % predicted), suggesting that CCL2 expression has a certain predictive value for pulmonary function in patients with IPF.

Alpha-defencins include antimicrobial and cytotoxic peptides that human neutrophils contain in azurophil granules and belong to the mammalian neutrophilic peptide family, and their level has been shown to be elevated in the serum of patients with SSc and is associated with lung involvement^30,31^. Our GSEA results revealed enrichment in azurophil granule, and the scRNA-seq results revealed that the CCL2 expression level was upregulated in several cell types including alveolar epithelial type 1 cell, alveolar epithelial type 2 cell, mast cell, ciliated cell, club cell, fibroblast, M1 macrophage, M2 macrophage, monocyte and plasma cell. These results show that in these cell types of IPF patients, alpha-deficiency-related immune reactions might be activated. Mukae’s study also revealed that plasma alpha-defensin levels were negatively correlated with arterial oxygen tension (PaO2), lung function [vital capacity (%VC), forced expiratory volume in 1 s (FEV1), and carbon monoxide transfer factor (%TLCO)] in IPF patients, indicating that alpha-defencins play important roles in the pathogenesis of IPF^30^. We speculate that CCL2 and alpha-defencins interact to mediate the pathogenesis of IPF.

In SSc patients, the GSEA results revealed enrichment of various fatty acid metabolic processes, such as acyl − CoA metabolic process, cholesterol biosynthetic process, fatty − acyl − CoA biosynthetic process, fatty acid synthase activity and fatty acid metabolism, etc. These results indicate that CCL2 may play a role in the pathogenesis of SSc by affecting the metabolism of fatty acids. Studies have shown that adipose tissue and its secretion group significantly contribute to the pathogenesis of SSc. Adipocytes are directly involved in the pathogenesis of SSc through adipocyte-myofibroblast transformation (AMT) and are the source of myofibroblast precursors at different tissue sites [e.g., dermal white adipose tissue (dWAT) in the skin and lipofibroblasts in the lung]^32^. Moreover, fatty acid metabolism also plays a role in IPF pathogenesis. As mediators of profibrotic reactions, fatty acids and their metabolites (stearic acid, palmitic acid, arachidic acid, etc.) regulate the phenotypic changes in various lung cells during lung tissue maladaptation to remodelling^33^. Repetitive genetic or environmental stimulation of alveolar epithelial type 2 (AT2) cells can lead to endoplasmic reticulum (ER) stress, which can ultimately drive downstream fibrotic remodelling of IPF lungs, and fatty acid (FA) synthesis and composition are involved in ER stress^34,35^. Fatty acid oxidation (FAO) produces a large amount of ATP, provides energy to support the polarization of M2 macrophages, and activates the signalling pathway of macrophage polarization, enabling M2 macrophages to produce profibrotic mediators, such as TGF-β1, activate fibroblasts, and promote extracellular matrix (ECM) deposition^36^. Therefore, the extensive enrichment of fatty acid metabolism in SSc patients with high CCL2 expression suggests possible pulmonary fibrotic injury. In addition, according to our scRNA-seq results, compared with that in healthy donors, CCL2 expression was upregulated in these 3 cell types (alveolar epithelial type 2 cell, M2 macrophage and fibroblast), suggesting that fatty acid metabolism plays a role in the pathogenesis of these 3 cell types in IPF patients.

One study revealed that the activation of CCL2/CCR2 signalling is involved in the pathogenesis of IPF through the recruitment of macrophages to the lung parenchyma and polarization of the M1 phenotype^37^. Our scRNA-seq results revealed that CCL2 expression in M1 and M2 macrophages from patients with IPF was significantly greater than that in healthy donors, suggesting a potential connection between CCL2 and macrophages in the pathogenesis of IPF.

Our results revealed that CCL2 expression was downregulated in dcSSc patients after 3 months of MMF treatment according to our result. Although this result does not prove a causal relationship between MMF treatment and the downregulation of CCL2 expression, it suggests that MMF may improve SSc by inhibiting the chemokine system, providing a possible direction for the treatment of SSc and even SSc-ILD.

Our study has several limitations. Our study used data from a public database and lacked support from clinical data. Some datasets have small sample sizes, and our study was limited to the transcriptome level, therefore, the results need to be further verified by basic experiments and prospective clinical trials. Further studies are needed to elucidate the potential mechanism of CCL2 in the pathogenesis of IPF and SSc.

## Materials and methods

### Data Collection

Figure 1 shows the flow chart of the study.

The Series Matrix File data file of GSE70866 (platforms: GPL14550 and GPL17077) was downloaded from the Gene Expression Omnibus (GEO) database (http://www.ncbi.nlm.nih.gov/geo/ ), which is from the donors’ bronchoalveolar lavage (BAL) cells and includes 3 cohorts: Freiburg (62 IPF patients and 20 healthy controls), LEUVEN (64 IPF patients), and SIENA (50 IPF patients). The whole GSE70866 expression data was obtained via batch correction and normalization of 3 cohorts via the “removeBatchEffect” function and the “normalizeBetweenArrays” function of the “limma” package in R software 4.3.2^38^. Before and after batch correction and normalization, PCA was conducted via the PCA function of the “FactoMineR” package version 2.11^39^.

The GSE181549 series matrix file data file (platform: GPL13497), which was obtained from the donors’ skin, was downloaded from GEO. A total of 113 SSc patients and 44 matched healthy controls were included in this dataset; 105 SSc patients had a 2nd biopsy, 76 patients had a 3rd biopsy, and 1 patient had a 4th biopsy. We chose 1st biopsy patients and healthy controls for the following study.

5 other datasets of IPF (GSE27957, GSE28042, GSE93606, GSE32537, and GSE122960) and 3 datasets of SSc (GSE181549, GSE58095, and GSE97248) were also downloaded from GEO. The baseline IPF patients’ samples of GSE27957, GSE28042 and GSE93606 was removed batch effect and standardization based on the “removeBatchEffect” function and the “normalizeBetweenArrays” function of “limma” package and of in R software^38^.

Table 1 shows the information of all the datasets used in this study.

**Table 1:** The information of all the datasets used in the study.

GEO Series	Platform	Tissue	Sample		Type
			IPF	Control	
GSE70866	GPL14550,GPL17077	Bronchoalveolar lavage (BAL) cells	176	20	mRNA
GSE27957	GPL5175	Peripheral blood mononuclear cell (PBMC)	45	0	mRNA
GSE28042	GPL6480	PBMC	75	19	mRNA
GSE93606	GPL11532	Peripheral whole blood	154 (57 baseline)	20	mRNA
GSE32537	GPL6244	Lung	119	50	mRNA
GSE122960	GPL20301	Lung	4	8	scRNA
			**SSc**	**Control**	
GSE181549	GPL13497	Forearm skin	295 (113 1st biopsy)	44	mRNA
GSE58095	GPL10558	Skin	61	36	mRNA
GSE97248	GPL14550	Skin	36 [diffuse cutaneous systemic sclerosis (dcSSc), before and after using MMF]	0	mRNA

### WGCNA and key module genes identification

WGCNA was performed on the GSE70866 and GSE181549 datasets via the “WGCNA” package in R software to screen the gene modules^40^. First, the median absolute deviation (MAD) of each gene was determined, and 25% of the genes with the smallest MAD and genes whose MAD was less than 0.01 were removed. On the basis of the correlation coefficient R^2^ > 0.85, the “picksoftthreshold” function was used to construct the gene expression patterns of scale-free networks. After the modules are acquired, according to the module of the first principal component from different module eigengenes (ME), the ME is associated with the clinical features of the evaluation module–trait relationships. The modules with the most significant positive and negative correlations of the module-trait relationships were subsequently screened. Finally, the module membership (MM) and gene significance (GS) scores of the modules were also assessed to account for module significance (MS). The first two modules of both datasets were chosen for the following study.

### Construction of a PPI network

The intersecting genes of these 2 groups of modules were then input into the STRING database, and Cytoscape 3.10.0 software was subsequently used to visualize a PPI network. By using the MCC method of the cytoHubba plug-in, the first-ranked 10 genes were chosen for the following study^41^.

### Survival and prognostic analysis

Survival information from the GSE70866 dataset was used to analyse the effects of the above 10 genes on the prognosis of patients with IPF. K–M curves of these 10 genes were generated via the “survminer” package (https://cran.r-project.org/web/packages/survminer/index.html , v.0.4.9) in R software, and the difference in survival between groups was statistically tested via the log‒rank method. Univariate Cox regression of these 10 genes was performed via the “survival” package (https://github.com/therneau/survival, v.3.6–4) and was visualized via the “forestploter” package (https://github.com/adayim/forestploter, v.1.1.2) of R software.

### Machine learning

Three machine learning algorithms (LASSO-Cox regression, SVM-RFE and Random Forest) were employed in the baseline IPF samples of GSE70866 to identify potential prognostic biomarkers from a set of 10 genes identified in the PPI network. LASSO regression, SVM-RFE and random forest algorithms were run on SSc and control samples from GSE53845 to screen for potential SSc diagnostic biomarkers. LASSO-Cox and LASSO regression can improve prediction accuracy and minimize the risk of overfitting by selecting variables^42^. The random forest model is an effective prediction tool with high accuracy, sensitivity and specificity, is independent of variable conditions, and can help assess the importance of variables^43^. SVM-RFE enables variable selection and interpretation of associations between predictive and response variables when analysing biomedical data and is achieved with high levels of accuracy and speed and low computational costs^44^. These 3 algorithms were conducted according to the R packages “glmnet”, “randomForest” and “e1071” (https://github.com/cran/e1071)^45,46^. The genes common to these algorithms are considered potential central genes for the prognosis of IPF and the diagnosis of SSc.

### Single-cell RNA data processing and clustering

The raw data of GSE122960 was downloaded from the GEO database for single-cell sequencing analysis. 4 IPF samples (GSM3489183, GSM3489184, GSM3489188, and GSM3489190) and 4 healthy samples (GSM3489182, GSM3489185, GSM3489189, and GSM3489191) were chosen according to the donors’ ages^47^. The 8 samples of GSE122960 were analysed according to the “Seurat” package version 5.1.0^48^. The cells were filtered with the criteria of 200 < number of feature RNA < 5000, percent of mt < 20, number of genes in single cell < 30,000, percentage of ribosomal genes > 3 and percentage of hemoglobin genes < 0.1. The FindVariableFeatures function was used, and the top 2000 hypervariable genes were selected on the basis of the variance stabilization transformation (vst) method. The top 2000 genes selected previously were scaled by using the ScaleData function, and the RunPCA function was subsequently used to reduce the dimension of the PCA. Dim = 1:15 was chosen for the following analysis. The IntegrateLayers function and HarmonyIntegration method in the “Seurat” package version 5.1.0. were subsequently used to remove the batch effect between samples^48^. The cells were then clustered into 27 clusters via the FindNeighbors and FindClusters functions with a resolution = 0.8. We used the RunUMAP function for visualization, and the cell type annotation was based on the CellMarker2.0 database (http://117.50.127.228/CellMarker/), Sun et al.’s study and the original article of GSE122960^47,49^.

### Enrichment analysis

GSEA is a reliable enrichment method based on gene expression levels^50^. In our research, we calculated the cut-off level of CCL2 via the “survival” package on the basis of the gene expression level and prognostic information of the merged dataset of GSE27957, GSE28042, and GSE93606, and divided the IPF samples into 2 groups on the basis of the cut-off level. Additionally, the SSc samples in GSE58095 were divided into 2 groups on the basis of the median CCL2 expression level. We used the “ClusterProfiler” package for GO (Gene Ontology) and KEGG (Kyoto Encyclopedia of Genes and Genomes) analysis via GSEA enrichment analysis of these 2 datasets, and p values < 0.05 and FDRs < 0.25 were considered statistically significant^51–54^.

### Statistical analysis

The analyses and visualization in this research were performed in R software (version 4.3.2) and GraphPad Prism 9.5.0. The linear fit was performed via the Levenberg‒Marquardt method. Based on whether the data follows a normal distribution and whether it is a paired design, we used a t test or Mann‒Whitney U test or Wilcoxon matched-pairs signed-rank test to determine significant differences between the groups, and *p* < 0.05 was the criterion for statistical significance.

## Conclusion

Our study revealed the common characteristic gene CCL2 in IPF and SSc and its expression was negatively correlated with the survival and lung function of IPF patients. In addition, CCL2 might be a potential target of MMF in the treatment of dcSSc. The mechanism of CCL2 in the pathogenesis of IPF and SSc may be related to TGF-β pathway, chemokine system, alpha-defencins and fatty acid metabolic processes. Fibroblasts, monocytes, M1 macrophages and M2 macrophages may play a role in the pathogenesis of CCL2 in relation to IPF. Our study provides a possible direction for the pathogenesis and treatment of SSc and IPF.

## Data Availability

The datasets extracted and/or analysed during the current study are available in the GEO repository, (https://www.ncbi.nlm.nih.gov/geo/query/acc.cgi?acc=GSE70866, https://www.ncbi.nlm.nih.gov/geo/query/acc.cgi?acc=GSE27957, https://www.ncbi.nlm.nih.gov/geo/query/acc.cgi?acc=GSE28042, https://www.ncbi.nlm.nih.gov/geo/query/acc.cgi?acc=GSE93606, https://www.ncbi.nlm.nih.gov/geo/query/acc.cgi?acc=GSE32537, https://www.ncbi.nlm.nih.gov/geo/query/acc.cgi?acc=GSE122960, https://www.ncbi.nlm.nih.gov/geo/query/acc.cgi?acc=GSE181549, https://www.ncbi.nlm.nih.gov/geo/query/acc.cgi?acc=GSE58095, https://www.ncbi.nlm.nih.gov/geo/query/acc.cgi?acc=GSE97248).
